# Giant post-traumatic liponecrotic granuloma of the gluteal region: A case report with multimodality imaging findings

**DOI:** 10.1016/j.radcr.2026.07.070

**Published:** 2026-08-13

**Authors:** Chaimae Abourak, Yahya El Harras, Kaoutar Imrani, Ittimade Nassar, Yassine Hafiani

**Affiliations:** a Department of Radiology, Faculty of Medicine and Pharmacy, Azzamouri Hospital, Ibn Sina University Hospital, Mohammed V University, Kenitra, Morocco; b Central Radiology Department, Faculty of Medicine and Pharmacy, Ibn Sina University Hospital, Mohammed V University, Rabat, Morocco; c Department of Anesthesiology and Critical Care, Faculty of Medicine and Pharmacy, Azzamouri Hospital, Ibn Sina University Hospital, Mohammed V University, Kenitra, Morocco

**Keywords:** Liponecrotic granuloma, Fat necrosis, Magnetic resonance imaging, Post-traumatic lesion, Soft-tissue pseudotumor

## Abstract

Post-traumatic liponecrotic granuloma is a benign soft-tissue lesion resulting from adipose tissue necrosis after blunt trauma. Although post-traumatic fat necrosis is a recognized entity, large encapsulated lesions with a pseudotumoral appearance are rare and may closely mimic soft-tissue neoplasms, creating a diagnostic challenge. In this setting, multimodality imaging is essential for accurate lesion characterization and appropriate patient management. We report the case of a 40-year-old woman presenting with a progressively enlarging left gluteal mass that developed over 5 years following a road traffic accident. Clinical examination revealed a well-defined, firm, painless mass without overlying inflammatory skin changes. Ultrasound demonstrated a large avascular subcutaneous lesion measuring 122 × 109 × 89 mm (craniocaudal × anteroposterior × transverse). Computed tomography showed a well-circumscribed lobulated fat-containing mass with internal calcifications. Magnetic resonance imaging demonstrated a well-defined lobulated subcutaneous lesion with a thin hypointense capsule enclosing a central fatty component that was hyperintense on T1- and T2-weighted images, completely suppressed on fat-saturated sequences, and showed no significant postcontrast enhancement. The combination of a history of trauma and characteristic multimodality imaging findings strongly supported the presumptive diagnosis of post-traumatic liponecrotic granuloma. This case highlights the importance of correlating clinical history with ultrasound, computed tomography, and magnetic resonance imaging to accurately characterize chronic post-traumatic soft-tissue lesions. It also illustrates the diagnostic challenge posed by large encapsulated fat necrosis with a pseudotumoral appearance and emphasizes the value of multimodality imaging in establishing a confident presumptive diagnosis while potentially avoiding unnecessary invasive procedures.

## Introduction

Liponecrotic granuloma, also known as encapsulated fat necrosis, is a benign lesion resulting from a chronic inflammatory reaction of adipose tissue following adipocyte damage [1]. It may be associated with endogenous or exogenous lipids and primarily involves the subcutaneous tissue. This entity most commonly occurs after trauma, surgery, or local ischemia, leading to fat necrosis followed by a granulomatous inflammatory response and progressive fibrosis [1,2].

Clinically, it usually presents as a palpable subcutaneous mass, although most lesions remain small and asymptomatic. In some cases, however, encapsulated fat necrosis may progressively enlarge and present as a tumor-like mass months or even years after the initial triggering event [2]. Because of its variable clinical and radiological presentation, the diagnosis can be challenging and may mimic a soft-tissue neoplasm. Imaging, particularly ultrasound, computed tomography (CT), and magnetic resonance imaging (MRI), plays a key role in lesion characterization and diagnostic orientation.

We report a case of a giant post-traumatic liponecrotic granuloma of the gluteal region presenting as a slowly enlarging pseudotumoral soft-tissue mass. Rather than emphasizing the anatomical location itself, this report highlights the diagnostic challenge posed by an unusually large encapsulated lesion with imaging features overlapping those of soft-tissue neoplasms. It also illustrates the complementary value of ultrasound, CT, and MRI in supporting a confident presumptive diagnosis based on characteristic clinicoradiological findings, thereby helping to avoid unnecessary invasive procedures.

## Case report

A 40-year-old woman with no significant medical or surgical history presented with a progressively enlarging left gluteal mass that had evolved over 5 years following trauma. The patient reported being struck by a car as a pedestrian, with the point of impact involving the left buttock. Immediately after the accident, she experienced local pain associated with skin erythema. Over time, she gradually noticed the development of a painless, partially firm mass that progressively increased in size.

**Fig. 1:**
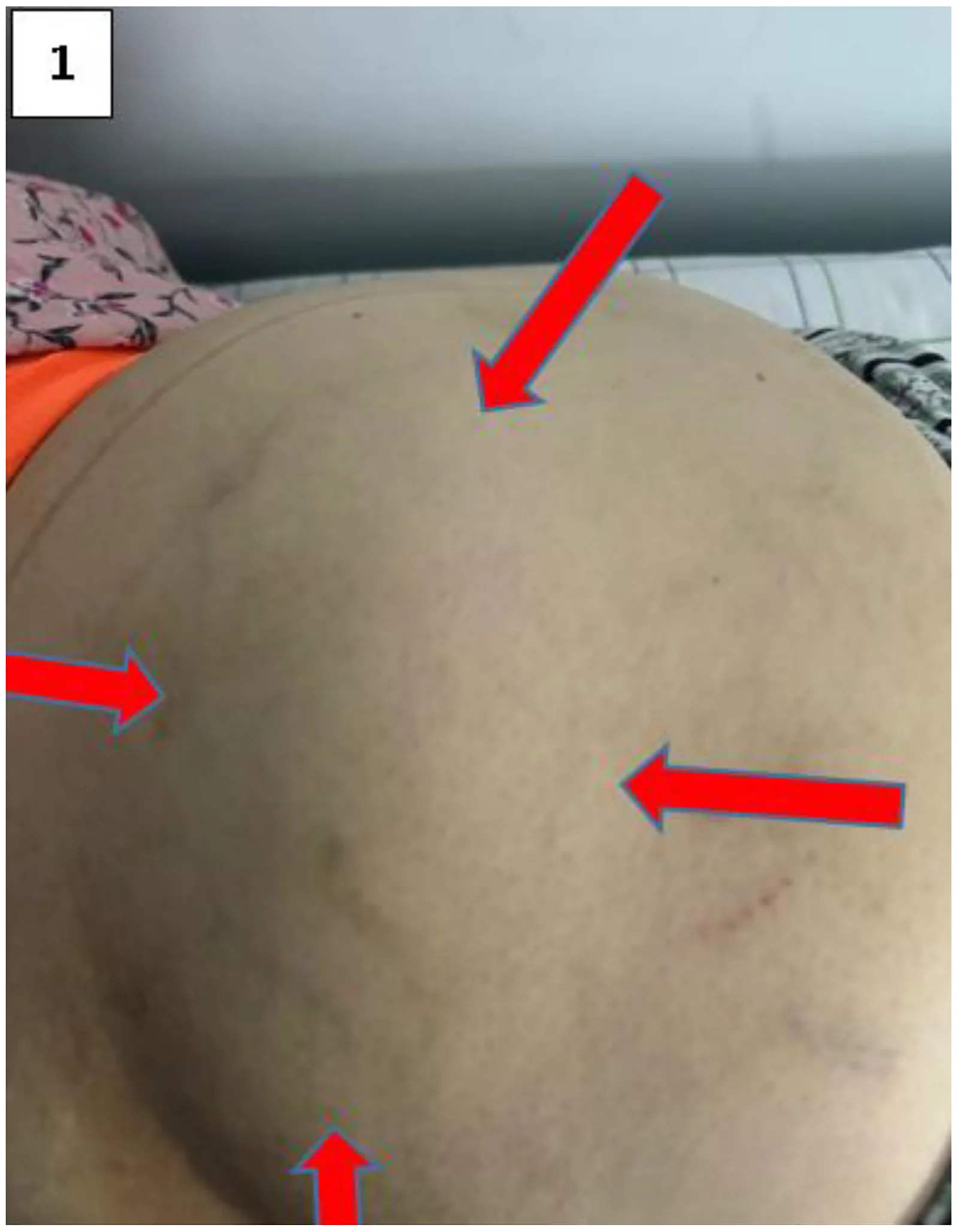
Clinical photograph of the left gluteal region showing a bulky, well-defined subcutaneous mass (red arrow) without overlying inflammatory skin changes.

Physical examination revealed a well-defined, relatively firm bulky mass located in the left gluteal region, without overlying inflammatory skin changes (Fig. 1). The remainder of the physical examination was unremarkable.

**Fig. 2:**
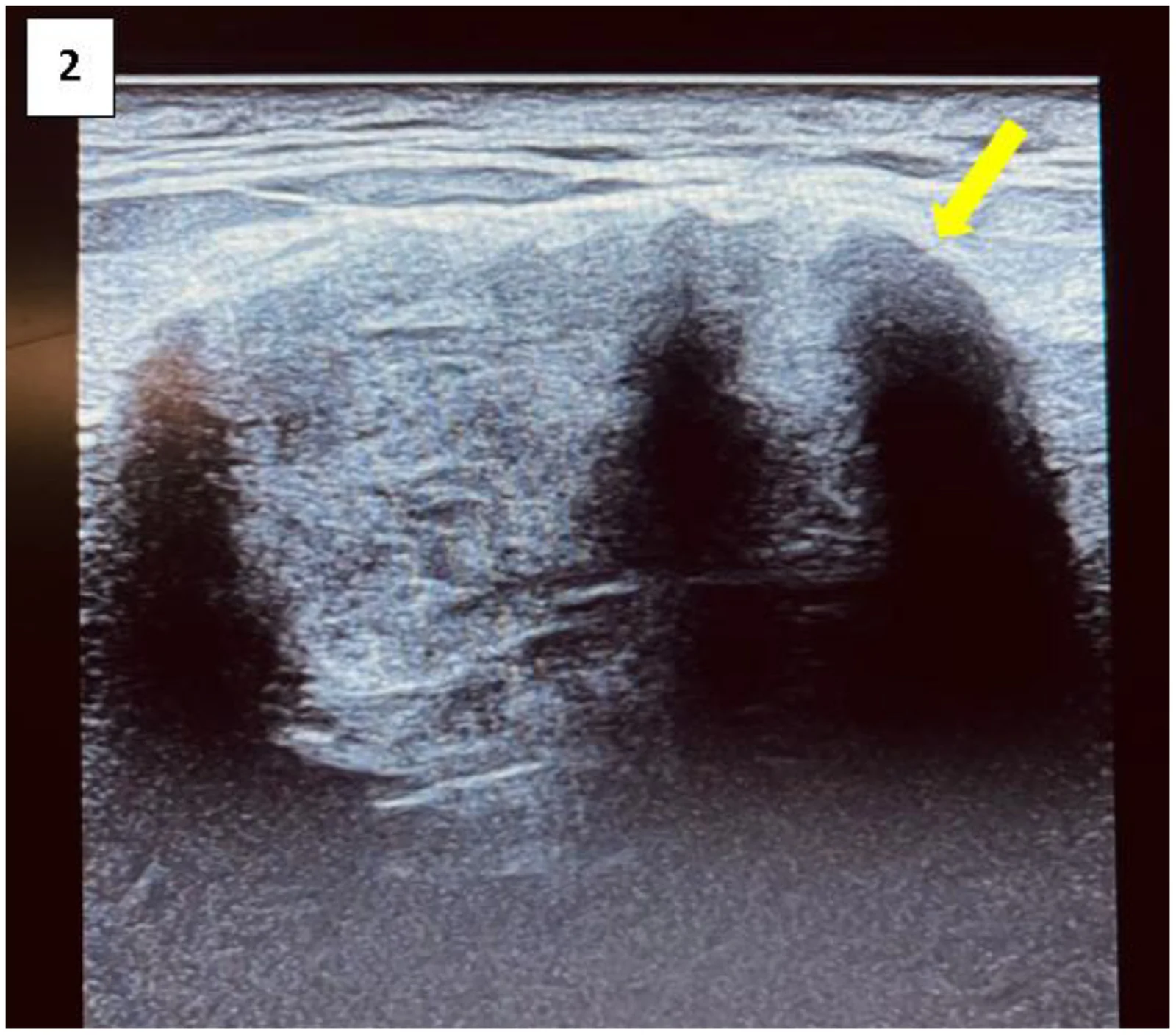
Transverse ultrasound image of the left gluteal soft tissues demonstrating a large, well-defined subcutaneous mass with lobulated margins and heterogeneous isoechoic echotexture, without internal vascularity on color Doppler. Peripheral calcifications (yellow arrow) produce posterior acoustic shadowing.

Soft-tissue ultrasound revealed a large, well-circumscribed subcutaneous mass with lobulated margins and heterogeneous isoechoic echotexture. Color Doppler examination showed no internal vascularity. Peripheral calcifications producing posterior acoustic shadowing were also identified (Fig. 2).

**Fig. 3:**
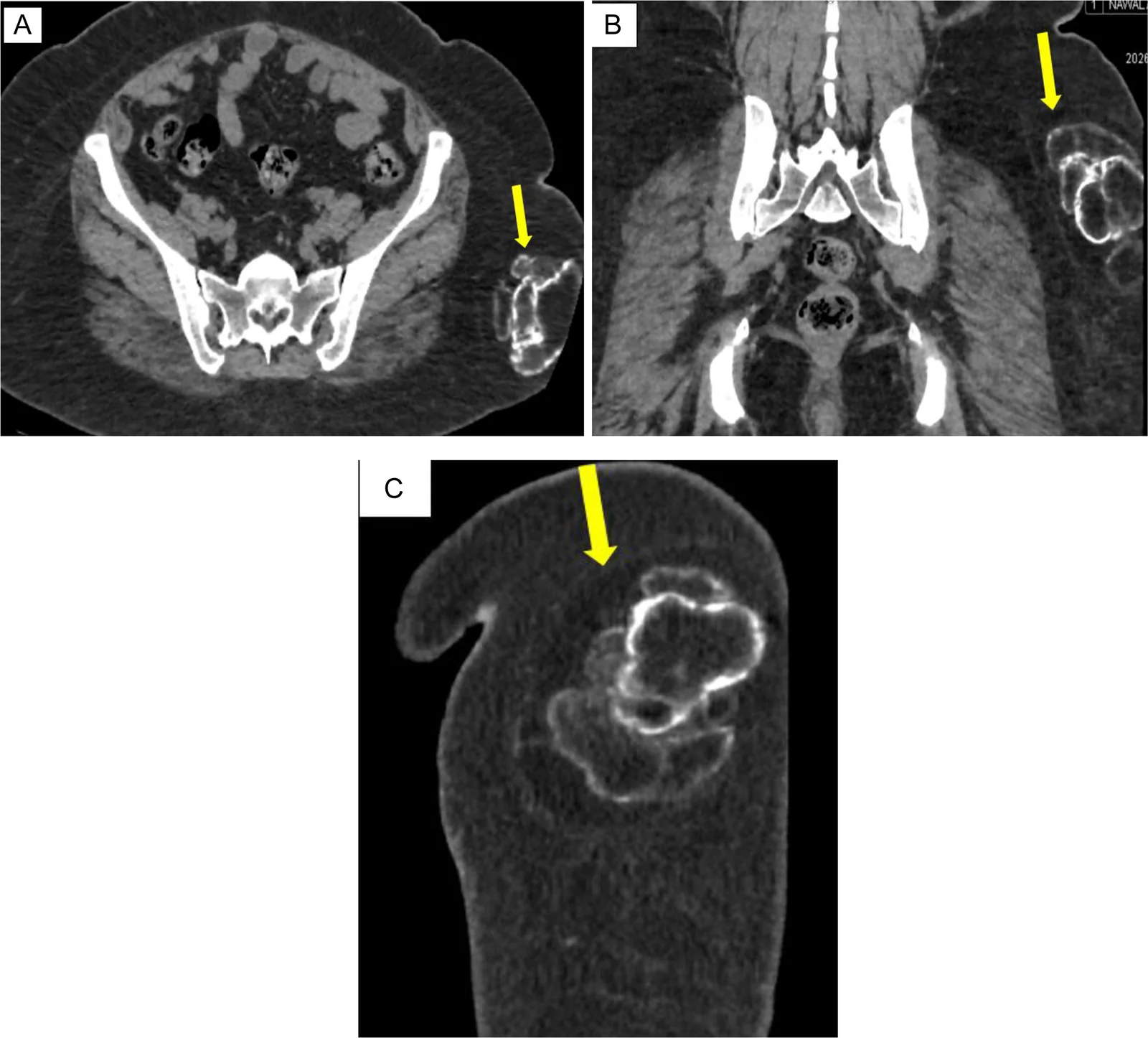
CT scan of the pelvis in soft-tissue window, axial (A), coronal (B), and sagittal (C) planes, showing a lobulated, well-circumscribed mass of fat density with internal calcifications, located within the subcutaneous soft tissues of the left gluteal region (yellow arrow).

A pelvic computed tomography (CT) scan was subsequently performed, demonstrating a large, well-defined lobulated fat-containing mass with a few internal calcifications within the subcutaneous soft tissues of the left gluteal region (Fig. 3).

**Fig. 4:**
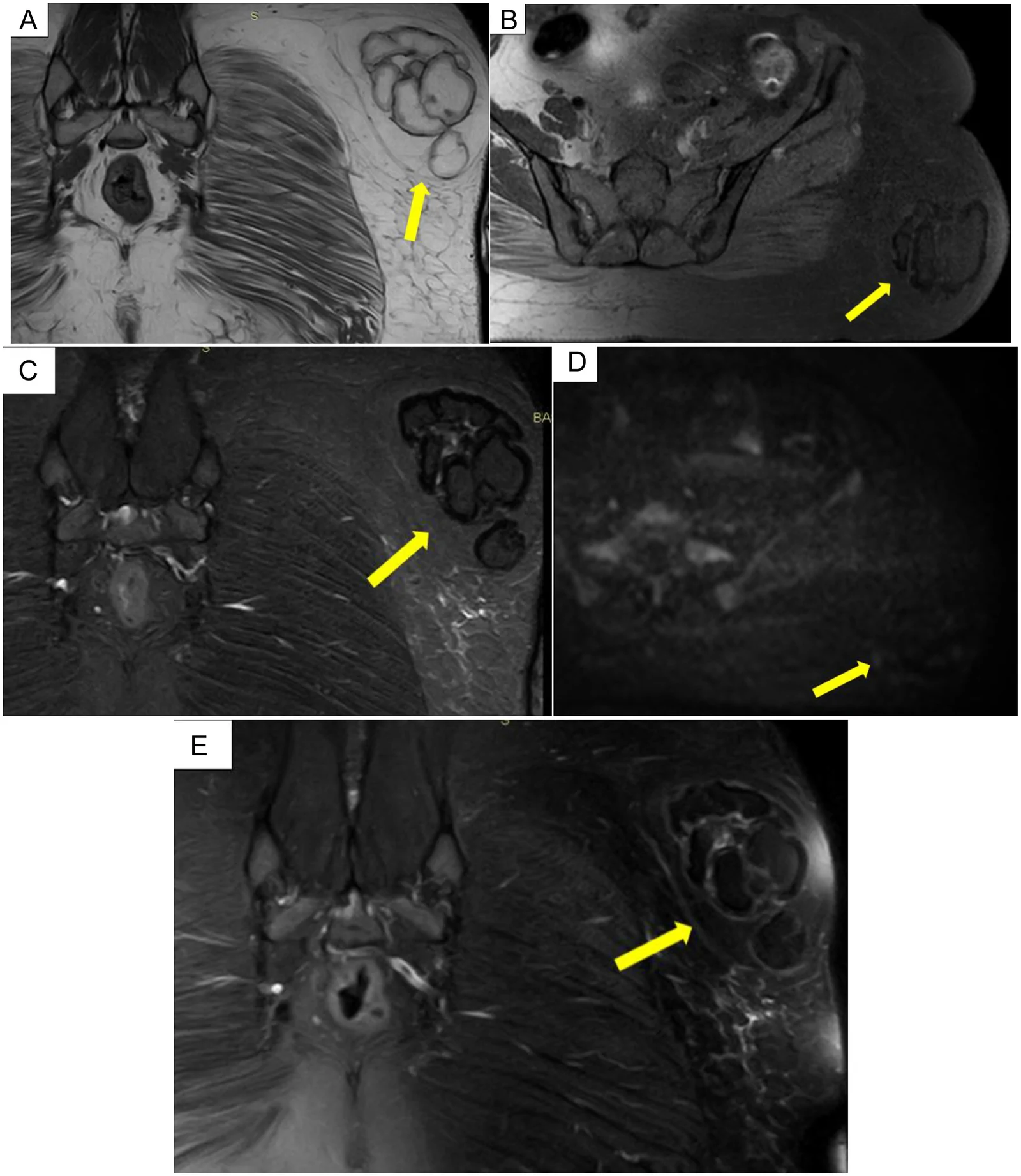
Pelvic MRI including coronal T1-weighted (A), axial T1 fat-saturated (B), coronal STIR (C), axial diffusion-weighted imaging (D), and contrast-enhanced coronal T1 fat-saturated (E) images, demonstrating a well-defined lobulated lesion confined to the subcutaneous tissue of the superolateral quadrant of the left gluteal region. The lesion is surrounded by a thin hypointense capsule visible on all sequences and contains a central fatty component that is hyperintense on T1-weighted images, with complete signal suppression on fat-saturated sequences, including STIR. No internal enhancement is observed after gadolinium administration (yellow arrow).

For further tissue characterization, pelvic magnetic resonance imaging (MRI) was performed. MRI revealed a well-circumscribed lobulated lesion confined to the subcutaneous adipose tissue of the superolateral quadrant of the left buttock, confined to the subcutaneous adipose tissue, measuring 122 × 109 × 89 mm (craniocaudal × anteroposterior × transverse). The lesion was surrounded by a thin capsule that appeared hypointense on all sequences and enclosed a central fatty component demonstrating high signal intensity on T1-weighted images, with complete signal suppression on STIR and other fat-saturated sequences. No significant enhancement was observed after gadolinium administration (Fig. 4).

The unusually large size of the lesion, its slow progression over 5 years, and its pseudotumoral appearance initially raised concern for a soft-tissue neoplasm. However, the patient's characteristic history of blunt trauma combined with the concordant multimodality imaging findings was highly suggestive of post-traumatic encapsulated fat necrosis (liponecrotic granuloma), making this the most likely diagnosis.

Given the characteristic clinical history and the typical imaging features demonstrated on ultrasound, CT, and MRI, a confident presumptive diagnosis of post-traumatic liponecrotic granuloma was established. As the imaging findings were considered highly characteristic and no radiological features suggestive of malignancy were identified, histopathological confirmation was not obtained.

Unfortunately, the patient was subsequently lost to follow-up; therefore, information regarding further management, histopathological confirmation, or imaging follow-up was not available.

## Discussion

Post-traumatic liponecrotic granuloma, also referred to as encapsulated fat necrosis, is a benign lesion resulting from aseptic degeneration of adipose tissue secondary to trauma. Small foci of post-traumatic fat necrosis are frequently encountered in routine clinical practice, particularly in areas exposed to blunt trauma, and are often asymptomatic or incidentally detected. In contrast, encapsulated lesions that progressively enlarge and present as pseudotumoral masses are uncommon and may pose a significant diagnostic challenge because of their resemblance to soft-tissue neoplasms. When histopathological examination is performed**,** this entity is characterized by a chronic nonallergic foreign-body reaction with persistent inflammation surrounding foci of necrotic adipose tissue. It has been described in patients of various ages but is reported more frequently in adolescents and middle-aged women, with a predilection for the lower limbs, although involvement of the trunk, hip, and upper limbs has also been reported [1,2]. The educational value of the present case lies not in its gluteal location itself, but in the unusually large size of the lesion, its prolonged evolution over 5 years, its pseudotumoral presentation closely mimicking a soft-tissue neoplasm, and the contribution of multimodality imaging to establishing a confident presumptive diagnosis.

The pathophysiology of liponecrotic granuloma remains incompletely understood. The most widely accepted mechanism involves local hemorrhage, vascular insufficiency, edema, and adipocyte ischemia following trauma, leading to aseptic fat necrosis with saponification under the action of tissue and blood lipases. Progressive inflammation subsequently induces a granulomatous reaction and fibrosis surrounding areas of necrotic adipose tissue. In some cases, interruption of the vascular supply results in infarction of fat lobules followed by encapsulation. Secondary degenerative changes, including fibrosis, dystrophic calcifications, and pseudomembrane formation, further contribute to the broad spectrum of clinical and imaging appearances [[3], [4], [5]].

Clinically, liponecrotic granuloma usually presents as a firm, slowly growing, palpable subcutaneous mass, although most lesions remain small. Less commonly, they progressively enlarge and simulate a soft-tissue neoplasm, particularly when they reach a considerable size, as observed in our patient. Associated findings may include local deformity, skin retraction, ecchymosis, or skin discoloration. Another characteristic feature is the long interval between the initial trauma and clinical presentation, which may extend over several months or even years. In our patient, the lesion became clinically evident 5 years after the traumatic event. Consequently, the causal trauma is often forgotten. Tsai et al. reported that only 23% of patients recalled a previous traumatic episode [2], further complicating the diagnosis and increasing the risk of confusion with soft-tissue tumors.

The imaging features of post-traumatic liponecrotic granuloma vary according to the stage of evolution, from acute fat necrosis to chronic encapsulation and fibrosis [2]. This temporal evolution explains the marked heterogeneity of imaging findings. In our patient, the lesion's unusually large size and pseudotumoral appearance required a systematic multimodality assessment, which proved essential for lesion characterization and exclusion of malignancy.

On ultrasound, fat necrosis usually appears as a heterogeneous lesion with variable echogenicity. It may present as peripheral hyperechoic areas surrounding central hypoechoic or heterogeneous components. In some cases, the lesion is iso- or hypoechoic, reflecting different stages ranging from acute inflammation to chronic fibrosis. Color Doppler typically demonstrates absent or minimal internal vascularity. In our patient, ultrasound demonstrated a large, well-circumscribed avascular isoechoic mass with posterior acoustic shadowing caused by peripheral calcifications.

Computed tomography is particularly useful for demonstrating the fatty nature of the lesion, dystrophic calcifications, and the presence of a thin fibrous capsule, findings that strongly support a benign diagnosis [4]. In our patient, CT demonstrated a well-defined lobulated fat-containing mass with internal calcifications, consistent with chronic encapsulated fat necrosis.

Magnetic resonance imaging provides optimal tissue characterization. Fat necrosis typically demonstrates high signal intensity on T1-weighted images, with complete signal suppression on STIR or other fat-suppressed sequences, reflecting its fatty composition, whereas the fibrous capsule appears hypointense on all sequences. Enhancement, when present, is usually limited to the capsule or inflammatory components and varies according to the stage of evolution [3]. In our patient, MRI clearly demonstrated these characteristic features and, together with the clinical history of trauma, strongly supported a presumptive diagnosis while excluding imaging findings suggestive of lipomatous malignancy.

Although no single imaging feature is pathognomonic, the combination of a characteristic history of trauma and concordant ultrasound, CT, and MRI findings can strongly suggest the diagnosis of encapsulated fat necrosis and reliably differentiate it from malignant soft-tissue tumors in typical cases. While histopathological examination remains the reference standard in diagnostically challenging cases or when malignancy cannot be confidently excluded, it is not systematically required. In patients with characteristic clinical and imaging findings, a confident presumptive diagnosis can often be established, allowing appropriate conservative management or therapeutic excision without prior biopsy.

A limitation of the present case is the absence of histopathological confirmation and clinical follow-up, as the patient was lost to follow-up after imaging evaluation. Consequently, the diagnosis should be regarded as presumptive, based on the characteristic clinical history and concordant multimodality imaging findings.

The differential diagnosis is broad and includes both post-traumatic and nontraumatic lesions. Among post-traumatic conditions, Morel-Lavallée lesion, chronic organized hematoma, and early-stage myositis ossificans should be considered [2]. Morel-Lavallée lesion is a closed degloving injury that typically develops within the potential space between the subcutaneous tissue and the deep fascia following blunt trauma. In contrast, the lesion in our patient was confined to the subcutaneous adipose tissue and demonstrated homogeneous fat signal intensity, a thin fibrous capsule, complete fat suppression, and no significant enhancement, findings that favored encapsulated fat necrosis over a Morel-Lavallée lesion [2,4]. Chronic organized hematoma was considered less likely because of the absence of blood degradation products, fluid–fluid levels, or progressive peripheral enhancement [2,4]. Other differential diagnoses include seroma, lipoma, epidermal inclusion cyst, dermatofibrosarcoma protuberans, and well-differentiated liposarcoma [2,4]**.** Well-differentiated liposarcoma was considered because of the lesion's large size and fat-containing appearance; however, the absence of thick or nodular enhancing septa, nonadipose soft-tissue components, internal vascularity, or other aggressive imaging features, together with the characteristic history of trauma, strongly favored post-traumatic encapsulated fat necrosis [2,4], described in several anatomical locations, including the breast [1], and the lower limbs [6].

The present case illustrates not the rarity of the anatomical location itself, but the diagnostic challenge posed by an unusually large encapsulated lesion with a pseudotumoral appearance closely mimicking a soft-tissue neoplasm. It further emphasizes the value of careful clinicoradiological correlation and multimodality imaging in supporting a confident presumptive diagnosis and helping to avoid unnecessary invasive procedures.

Regarding management, treatment is usually conservative. Small asymptomatic lesions may remain stable, regress, or resolve spontaneously [2]. Surgical excision is generally reserved for large symptomatic lesions or when diagnostic uncertainty persists despite imaging. In patients presenting with a characteristic clinical history and typical multimodality imaging findings, conservative management may be appropriate without prior histopathological confirmation.

Nevertheless, the absence of histopathological confirmation and imaging follow-up represents a limitation of the present report. Therefore, the diagnosis should be interpreted as presumptive, based on the characteristic clinical history and highly concordant multimodality imaging findings.

In most cases, no specific treatment is required, reflecting the benign and often self-limiting nature of this condition [2].

## Conclusion

Post-traumatic liponecrotic granuloma is a benign condition that may mimic soft-tissue tumors, particularly when presenting as a large encapsulated mass. Correlation of the clinical history with multimodality imaging findings, especially ultrasound, CT, and MRI, is essential to support a confident presumptive diagnosis and to distinguish this entity from malignant soft-tissue lesions. Although histopathological examination remains the reference standard when imaging findings are atypical or malignancy cannot be confidently excluded, characteristic clinicoradiological features may strongly suggest the diagnosis. The present case highlights the diagnostic challenge posed by giant encapsulated fat necrosis with a pseudotumoral appearance. The absence of histopathological confirmation and clinical follow-up should be acknowledged as a limitation of this report.

## Patient consent

Written informed consent for the publication of this case report was obtained from the patient.
